# Compact Microstrip Fixed-Frequency Double-Coupled Double-Tuned Filter with Selected Band Suppression

**DOI:** 10.3390/s25216768

**Published:** 2025-11-05

**Authors:** Dariusz Wójcik, Maciej Surma, Mirosław Magnuski

**Affiliations:** Department of Electronics, Electrical Engineering and Microelectronics, Silesian University of Technology, Akademicka 16, 44-100 Gliwice, Poland; maciej.surma@polsl.pl (M.S.); miroslaw.magnuski@polsl.pl (M.M.)

**Keywords:** microstrip filter, fixed frequency filter, double-tuned, double-coupled, band suppression

## Abstract

This paper presents the design and analysis of a compact microstrip fixed-frequency double-inductive-coupled filter with selected band suppression. The filter can be used as an input filter in wireless IoT sensors. The proposed structure has reduced dimensions and improved out-of-band attenuation, achieved through the use of radial stub lines as elements of the resonators. These lines act as capacitors within the passband, while in a selected sub-band as series resonant circuits, effectively enhancing attenuation. The frequency response of the filter is shaped using two transmission zeros: the first one improves the steepness of the frequency response at the upper transition band, while the second increases attenuation in a chosen sub-band of the stopband. An analysis of the filter is presented, and key equations describing its properties are derived. An example filter for the frequency band 2.391–2.525 GHz, with additional suppression introduced in the U-NII 5 GHz band was designed, manufactured and examined. The insertion loss achieved by the proposed filter is lower than 1.6 dB, its attenuation across the whole stopband exceeds 30 dB and reaches over 40 dB in the 4.7–5.9 GHz frequency band.

## 1. Introduction

The modern era of the Internet of Things (IoT) is characterized by a rapid increase in the number of sensors deployed in both industrial and consumer environments [1]. The IoT relies heavily on wirelessly connected sensors, which places high demands on radio transmission reliability. Wireless sensors often operate in highly disturbed environments with interference from other radio frequency and microwave devices and a high level of background noise produced by various electric and electronic devices. The ability of wireless sensors to operate in difficult radio conditions places high demands on the immunity of their receivers. In such conditions, bandpass filters play an important role in their front-end circuits. The primary function of the input filters is the suppression of out-of-band signals. These filters are also intended to eliminate spurious channels originating from frequency conversion in receivers with a non-zero intermediate frequency, as well as harmonic frequency conversion in non-zero and zero-IF receiver architectures [2]. Input filters with sufficiently high out-of-band attenuation increase receiver immunity to blocking by strong out-of-band interfering signals.

The input filter required in modern radiocommunication devices should feature low insertion loss in the passband, sufficiently high attenuation in the stopband, be easy to fabricate at low cost, as well as should have small electrical dimensions. Up to the VHF band fixed-frequency RF bandpass filters are most commonly built using lumped LC elements. Lumped element filters can have satisfactory parameters, provided that LC elements with a sufficiently high quality factor and an adequate low tolerance are used. The latter requirement often necessitates the use of tuned LC elements. Microwave filters, such as cavity filters, dielectric filters, helical filters, stripline filters, and microstrip filters, are constructed from distributed elements. A distinct category of bandpass filters is presented by SAW filters, which are manufactured for both RF and lower microwave frequencies. These filters can be smaller in size than cavity, dielectric, helical, stripline and microstrip filters; however, they exhibit higher insertion loss in the passband and lower stopband attenuation. Cavity, dielectric, and helical filters offer very good electrical performance but can have a large volume and require complicated fabrication methods. In contrast, microstrip and stripline filters are inexpensive, easy to fabricate, and manufactured using the same technological process as printed circuit boards, which significantly simplifies their integration with other circuits. Well-designed planar filters can be electrically small, making them a convenient solution for modern miniaturized radiocommunication devices.

In the case of microstrip filters, several types of resonators can be employed to reduce the electrical dimensions of the entire filter. Commonly used resonators with reduced dimensions include stepped-impedance resonators (SIRs), dual- and multi-mode resonators, or open-loop resonators (OLRs). SIRs enable smaller sizes at the desired operating frequencies by adjusting the impedance ratios and electrical lengths [3,4,5,6,7,8,9,10,11,12,13,14]. Dual and multi-mode resonators integrate multiple resonant modes into a single physical structure, effectively reducing the overall size and often halving the number of resonators required for a given filter order [5,6,13,15,16,17,18]. OLRs are compact due to their bent structures and the possibility of close coupling, which allows the resonant paths to occupy less space without degrading electrical performance [3,19,20,21,22,23,24,25,26,27]. The filter size can also be reduced using cross-coupling approaches, folding techniques, and multilayered structures. Cross-couplings can improve selectivity by generating TZs, and, if designed efficiently, can contribute to a reduced filter size by achieving the desired performance in a more compact layout [5,9,10,12,25,28,29,30,31,32]. Folding techniques employ resonator lines that are extensively folded to reduce their physical footprint. There are examples of doubly-folded half-wavelength hairpin lines and folded quarter-wavelength uniform impedance resonators [6,13,16,17,29,33,34]. Multilayer structures involve designing filters with overlapping components placed on different dielectric layers, which significantly reduce the overall circuit area [5,15,30,32,35,36].

**Figure 1:**
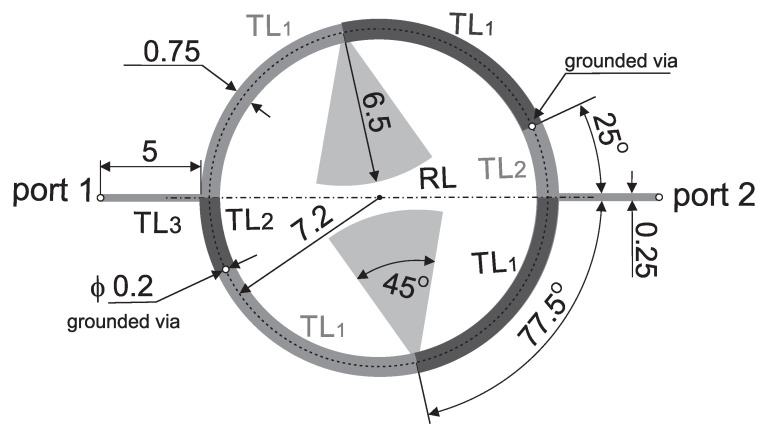
The layout of the compact microstrip fixed-frequency double-coupled filter with selected band suppression.

This paper presents the design and analysis of a compact microstrip fixed-frequency double-coupled double-tuned (CMFDCDT) filter with selected band suppression. The layout of the filter is shown in Figure 1. The proposed structure has reduced dimensions through the use of a multi-coupling technique combined with the implementation of folded resonators. Unlike the filters described in the previous work of the authors [37,38,39], the proposed solution incorporates resonators with radial stubs, which act as capacitors within the passband. This approach enables a reduction in the physical size of the resonators. Additionally, the radial stubs function similarly to series resonant circuits, effectively enhancing attenuation in a selected section of the stopband. The filter frequency response is shaped by two transmission zeros: the first improves the steepness of the frequency response in the upper transition band, while the second increases the attenuation in a targeted segment of the stopband. The design and evaluation of a sample filter operating in the ISM 2.4 GHz band, with additional suppression in the U-NII 5 GHz band, are also presented. During the design process, both circuit and full-wave simulations were used to verify the filter’s performance. Circuit simulations were performed using AWR Cadence ver. 17 [40] and full-wave simulations were performed using CST Studio Suite 2024 [41]. The filter insertion loss is below 1.6 dB within the 2.391–2.525 GHz band. Out-of-band attenuation exceeds 30 dB, reaching over 40 dB in the 4.7–5.9 GHz frequency range.

## 2. Microstrip Circular Double-Coupled Double-Tuned Filter

**Figure 2:**
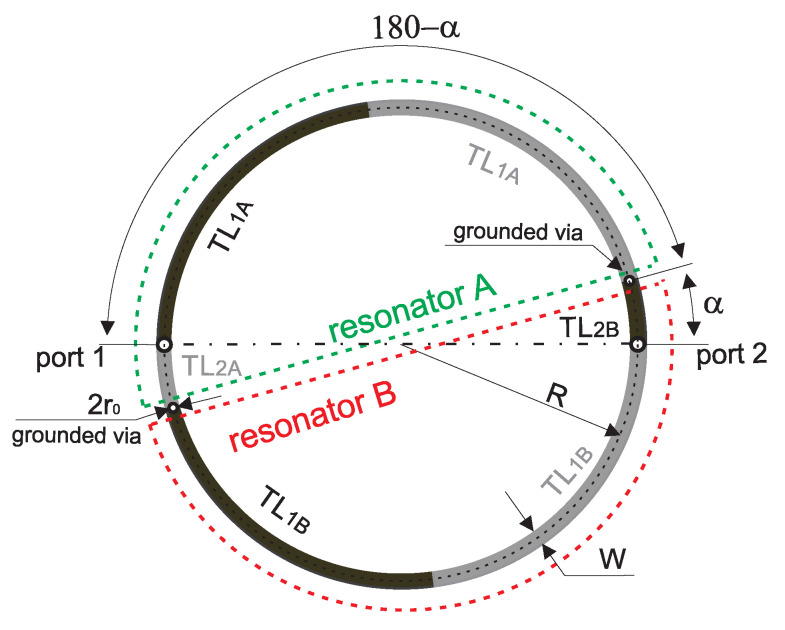
Layout of the initial, circular double-coupled double-tuned filter.

**Figure 3:**
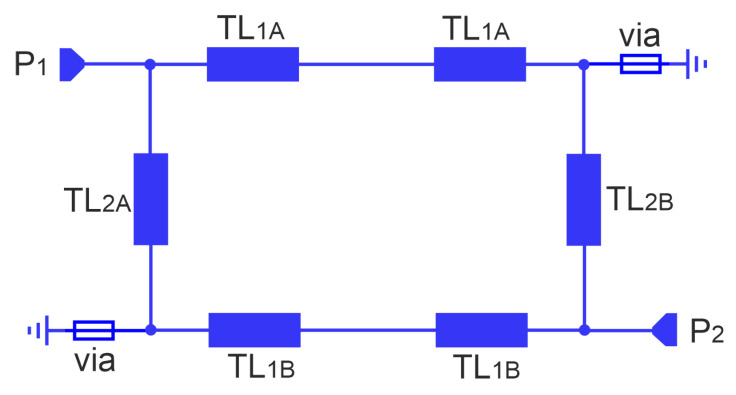
Equivalent circuit of the initial, circular double-coupled double-tuned filter.

In this section, the presentation of the proposed filter begins with an analysis of the initial microstrip circular double-coupled double-tuned filter which is a simplified version of the CMFDCDT filter deprived of radial RL and feeding TL_3_ lines. Its layout and equivalent circuit are presented in Figure 2 and Figure 3, respectively. The filter consists of resonators A and B. Each resonator is built of two sections of the TL_1*A*_/TL_1*B*_ line, a section of the TL_2*A*_/TL_2*B*_ line, and two grounding vias. The resonators connected together form a circular shape. The connections between lines TL_1_ and TL_2_ are grounded using vias or are fed from ports 1 and 2. The resonators are coupled through the common inductances of the vias.

In theory, such a microstrip filter has an infinite number of passbands. Their approximate center frequencies are given by

(1) $$f_{0}=\frac{nc}{2\left(2l_{1}+l_{2}+2\Delta l_{via}\right)\sqrt{\epsilon_{reff}}}\;n=1,2,3\ldots$$

describing the resonance frequencies of unloaded and uncoupled resonators. In Equation (1), *c* denotes the speed of light in vacuum, $\epsilon_{reff}$ is an effective dielectric constant, $l_{1}$ is the length of each section of the TL_1_ line, $l_{2}$ is the length of TL_2_ line and $\Delta l_{via}$ is the length of the equivalent line having the same inductance as the via

(2) $$\Delta l_{via}=\frac{L_{via}}{L_{l}}=\frac{L_{via}c}{Z_{0}\sqrt{\epsilon_{reff}}}$$

where $L_{l}$ is the inductance per unit length and $Z_{0}$ is the characteristic impedance of the line.

The inductance $L_{via}$ of the via having radius $r_{0}$ and height *h* was calculated according to [42]

(3) $$L_{via}=\frac{\mu_{0}}{4\pi }\left[h\ln\left(\frac{h+\sqrt{r_{0}^{2}+h^{2}}}{r_{0}}\right)+1.5\left(r_{0}-\sqrt{r_{0}^{2}+h^{2}}\right)\right]$$

where $\mu_{0}$ denotes the vacuum permeability.

The upper transition bands of the initial filter are shaped by transmission zeros located at frequencies

(4) $$f_{TZ1}=\frac{nc}{2\left(2l_{1}+\Delta l_{via}\right)\sqrt{\epsilon_{reff}}}\;n=0,1,2\ldots$$

For these frequencies, the sections of the TL_1_ line, together with the grounding via, are electrically half-wavelength; therefore, the interconnection node of TL_1_ and TL_2_ is grounded. A similar effect occurs at frequencies at which the TL_2_ line section, together with the grounding via, is half-wavelength

(5) $$f_{TZ2}=\frac{nc}{2\left(l_{2}+\Delta l_{via}\right)\sqrt{\epsilon_{reff}}}\;n=0,1,2\ldots$$

However, these frequencies are usually located beyond the useful operating band of the filter.

**Figure 4:**
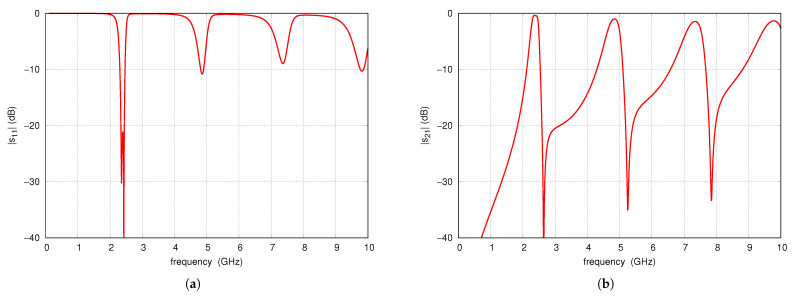
Full-wave simulation results of S-parameters of the example initial filter: (**a**) $s_{11}$ (**b**) $s_{21}$.

An example filter was designed adopting the RO4003C laminate with a dielectric constant of 3.55, a loss tangent of 0.0021, and a thickness of 0.813 mm. The scattering parameters of the filter designed for the 2.4 GHz band are shown in Figure 4 for the frequency range from 100 MHz to 10 GHz. The filter has a radius of R=11.8 mm, line widths of 1 mm, an angle α=15.5°, and a via diameter of 0.2 mm. According to Formulas (1) and (4) a center frequency is 2.429 GHz and the first TZ frequency is 2.69 GHz. According to full-wave simulations, the center frequency of the filter is 2.42 GHz, and the transmission zero is 2.65 GHz. The computations and simulations are at these points consistent. Subsequent passbands and TZs are located near the corresponding multiples of these frequencies. The lowest frequency $f_{TZ2}$, estimated from Formula (5), is approximately 24.9 GHz.

### Parametric Study of the Double-Coupled Double-Tuned Initial Filter

**Figure 5:**
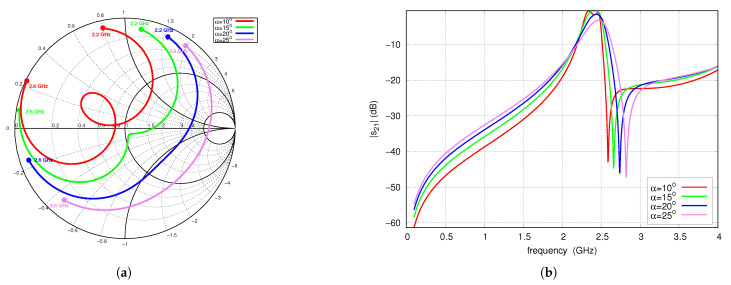
S-parameters vs. angle α, full-wave simulations: (**a**) $s_{11}$ (2.2–2.6 GHz frequency range), (**b**) $s_{21}$.

In this section, a parametric study is carried out to identify the impact of the initial filter components on its frequency response. Figure 5 shows the influence of the α angle on the S-parameters. Increasing α corresponds to an increase in the length of the TL_2*A*_ and TL_2*B*_ lines and a simultaneous decrease in the length of TL_1*A*_ and TL_1*B*_. An increase in α has little effect on center frequency and operating bandwidth but shifts $f_{TZ1}$ toward higher frequencies, which results from the shortening of the TL_1_ line. Changing α, which corresponds to moving the position of the resonator feed point, affects its matching conditions. For α values of 10° to 15°, an improvement in $s_{11}$ is observed, approaching an almost optimal value. For α values of 20° and 25°, $s_{11}$ deteriorates significantly.

**Figure 6:**
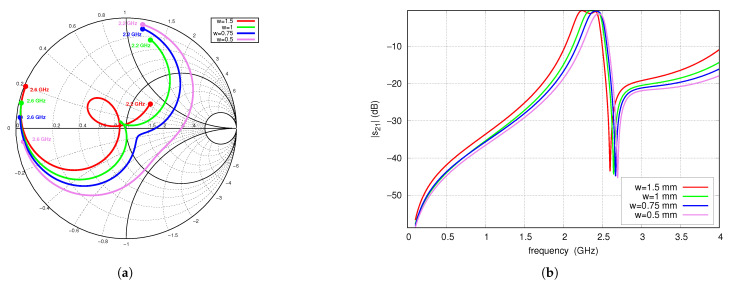
S-parameters for variable lines width *W*, full-wave simulations: (**a**) $s_{11}$ (2.2–2.6 GHz frequency range), (**b**) $s_{21}$.

Figure 6 shows the frequency behavior of the S-parameters for several values of the TL_1_ and TL_2_ line width *W*. Narrowing the width of the TL_1_ and TL_2_ lines increases the line impedance, which corresponds to an increase in inductance per unit length. Consequently, the constant inductance of the via, represented as the equivalent length $\Delta l_{via}$, has a decreasing influence on the center frequency and $f_{TZ1}$. A change in line width also affects the input impedance of the filter, the operating bandwidth, and the shape of the $s_{21}$ characteristics. For line widths of 0.5 mm and 0.75 mm, the impedance within the bandwidth is capacitive, for 1 mm the best impedance matching occurs and for 1.5 mm it is inductive.

**Figure 7:**
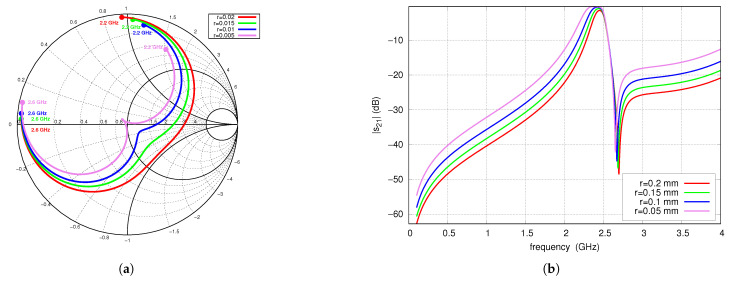
S-parameters vs. radius of the via $r_{0}$, full-wave simulations: (**a**) $s_{11}$ (2.2–2.6 GHz frequency range), (**b**) $s_{21}$.

Figure 7 shows the S-parameters as a function of frequency for several values of the via radius $r_{0}$. The decreasing of the via radius leads to an increase in the corresponding equivalent inductance. Therefore, reducing the via radius increases the coupling coefficient between the resonators, resulting in a widening of the operating band, a change in matching, and a decrease in attenuation occurring in the stopband. In the case of the filter shown, increasing the coupling coefficient improves the matching. Reducing the radius of the via slightly lowers the center frequency.

## 3. Microstrip Double-Coupled Filter with Selected Band Suppression

**Figure 8:**
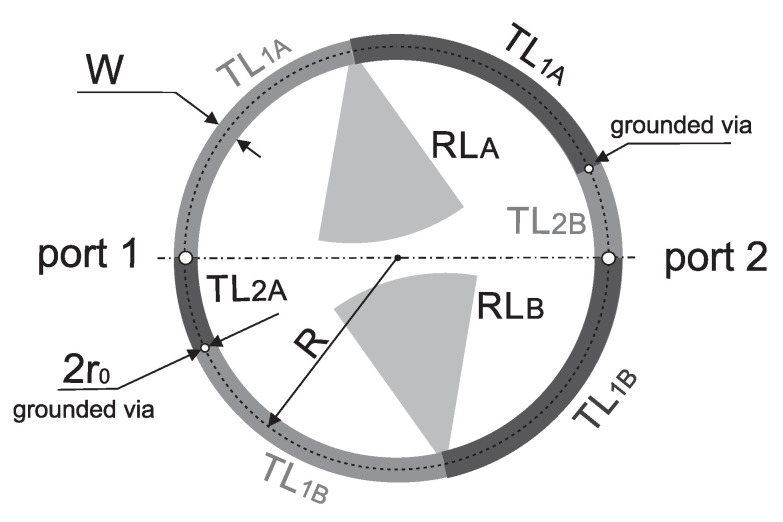
The layout of compact microstrip fixed-frequency double-coupled filter deprived of TL_3_ lines.

**Figure 9:**
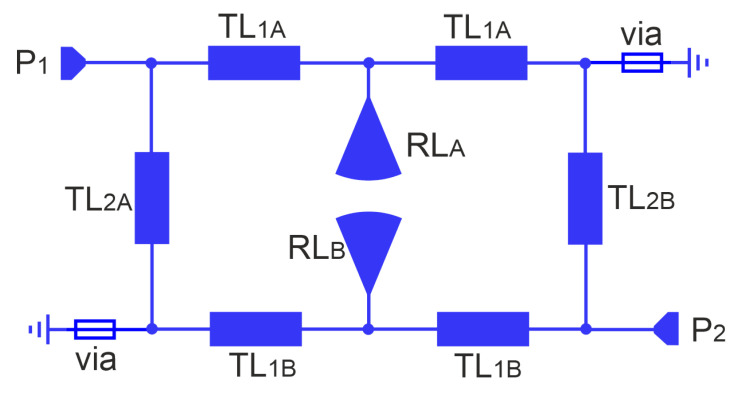
Equivalent circuit of the compact microstrip fixed-frequency double-coupled filter deprived of TL_3_ lines.

This section presents the properties of the CMFDCDT filter deprived of TL_3_ lines. The layout and circuit model are shown in Figure 8 and Figure 9, respectively. As will be demonstrated, its circuit can be reduced in the vicinity of the $f_{TZ1}$ frequency to the initial filter with modified TL_1*A*_ and TL_1*B*_ line lengths.

**Figure 10:**
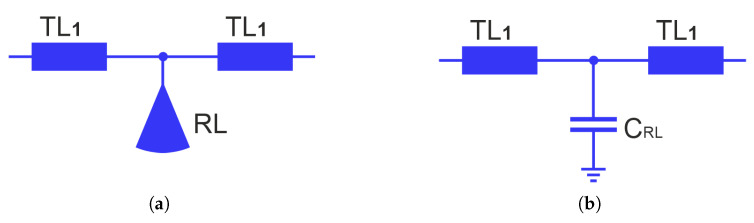
A part of the resonator formed by two sections of the TL_1_ line and RL radial line (**a**) and its the equivalent circuit model (**b**).

In order to determine the center frequency of the filter, a modal analysis of the lossless two-port network, which is part of the resonators and formed by two sections of the TL_1_ line and the RL radial line (see Figure 10a), is performed. For the purposes of the analysis, it is assumed that at the frequency of the first transmission zero $f_{TZ1}$, the input impedance of the radial line is Z_*RL*_. At this frequency and in its vicinity, neglecting losses, the radial line can be replaced by a capacitor with capacitance C_*RL*_.

(6) $$C_{RL}\approx \frac{1}{2\pi f_{TZ1}\left|Z_{RL}\right|}$$

**Figure 11:**
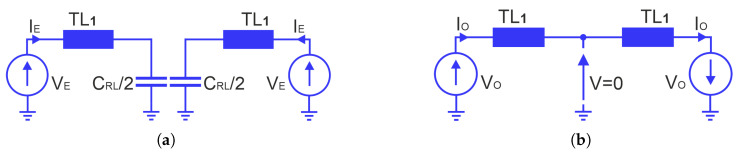
Circuit models for even (**a**) and odd (**b**) modes of the circuit from Figure 10.

Therefore, the equivalent circuit of the considered network can be represented by the circuit shown in Figure 10b. Subsequently, an even- and odd-mode analysis of the equivalent two-port network is carried out. The corresponding circuits of the even- and odd-mode are shown in Figure 11. As can be seen, $Y_{o}$ admittance is the same as that of a circuit without the capacitor, while the $Y_{e}$ admittance corresponds to the input admittance of the TL_1_ line loaded with a capacitance of C_*RL*_/2.

In the next step, the loading capacitance can be replaced by line sections that extend the TL_1_ lines by a length

(7) $$\Delta l_{RL}\approx \frac{\lambda \arctan(2|Z_{RL}|/Z_{0})}{2\pi }$$

**Figure 12:**
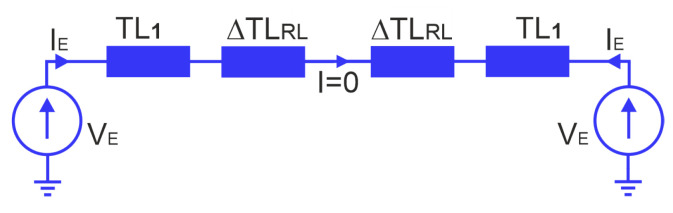
Substitute model for even mode with $\Delta TL_{RL}$ lines of the circuit from Figure 10.

**Figure 13:**
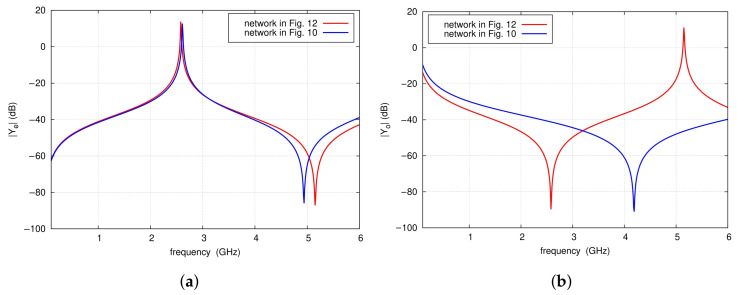
$Y_{e}$ and $Y_{o}$ circuit simulation results of the networks shown in Figure 10 and Figure 12.

For $2\left|Z_{RL}\right|\ll Z_{0}$ it is equivalent to

(8) $$\Delta l_{RL}\approx \frac{C_{RL}}{C_{l}}=\frac{C_{RL}cZ_{0}}{\sqrt{\epsilon_{reff}}}$$

where $C_{l}$ is the capacitance per unit length of the line. As a result, the even-mode substitute circuit shown in Figure 12 is obtained. Exemplary calculations are performed for the two-port network that is part of the filter shown in Figure 9 at the frequency $f_{TZ1}$ = 2.7 GHz, for the input impedance of the radial line of 50.3 Ω, and the equivalent capacitance C_*RL*_ of 1.15 pF. The $\Delta TL_{RL}$ section length is 7.32 mm. The graphs in Figure 13 present the frequency characteristics of the $Y_{e}$ and $Y_{o}$ admittance magnitudes of the network shown in Figure 12 in the vicinity of the center and first TZ frequencies. As can be observed, both the two-port network and its equivalent model exhibit $Y_{e}$ admittance magnitudes that identically tend toward infinity at frequency $f_{TZ1}$. However, the frequency characteristics of $Y_{o}$ of the proposed model deviate from those of the original network. The elements of the admittance matrix can be expressed by means of $Y_{e}$ and $Y_{o}$ according to:

(9) $$Y_{11}=Y_{22}=\frac{Y_{e}+Y_{o}}{2}\;\;Y_{21}=Y_{12}=\frac{Y_{e}-Y_{o}}{2}.$$

The equivalent model of the network, including the radial line, should provide a close approximation of its admittance matrix elements. It is important to note that for frequencies near $f_{TZ1}$, the dominant component is the admittance $Y_{e}$, whose magnitude tends to infinity, while $Y_{o}$ remains finite. Therefore, $Y_{o}$ can be omitted, which results in simplified formulas:

(10) $$Y_{11}\approx Y_{21}\approx \frac{Y_{e}}{2}$$

**Figure 14:**
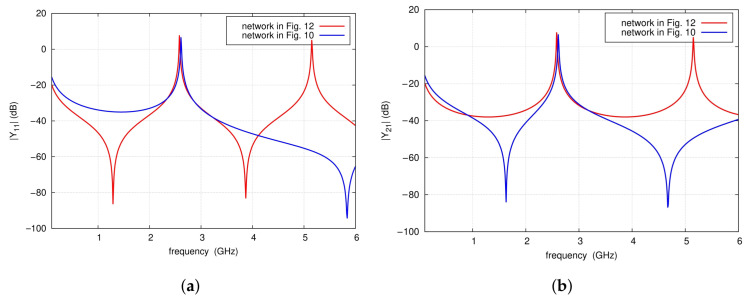
Y-parameters circuit simulation results of the networks shown in Figure 10 and Figure 12.

It follows that, in the vicinity of the frequencies $f_{TZ1}$ and $f_{0}$, the circuit in Figure 10a can be replaced by that of Figure 12, as the admittances $Y_{e}$ of both circuits have practically identical characteristics. In the analyzed example, the obtained model has sufficient accuracy of Y-parameters in the frequency range from 2.3 to 3.2 GHz (see Figure 14). For this model, the center frequency of the filter and the frequency $f_{TZ1}$ are given by the following formulas:

(11) $$f_{0}=\frac{c}{2\left(2\left(l_{1}+\Delta l_{RL}\right)+l_{2}+2\Delta l_{via}\right)\sqrt{\epsilon_{reff}}}$$

(12) $$f_{TZ1}=\frac{c}{2\left(2\left(l_{1}+\Delta l_{RL}\right)+\Delta l_{via}\right)\sqrt{\epsilon_{reff}}}$$

These formulas are modifications of Formulas (1) and (4), which describe the properties of the initial filter without radial lines, shown in Figure 2. The modifications introduced consist of taking into account the length of the $\Delta l_{RL}$, which replace the radial lines. The Formula (12) allows for verification of whether the assumed frequency of the first TZ matches the value obtained.

## 4. Proposed Filter Design

The derived formulas allow for the determination of the initial lengths of the TL_1_ and TL_2_ lines for the assumed center frequency and TZ frequencies. The procedure is as follows:

1. Select the dimensions of the radial line to obtain the assumed $f_{TZ_{RL}}$ frequency.
2. Determine the equivalent input impedance $Z_{RL}$ of the radial line for frequency $f_{TZ1}$.
3. Determine the lengths of $\Delta l_{RL}$ from formula (7).
4. Determine the initial length of the TL_1_ line to obtain the assumed value of $f_{TZ1}$, neglecting the influence of the via:
  (13) $$l_{1}\approx \frac{c}{4f_{TZ1}\sqrt{\epsilon_{reff}}}-\Delta l_{RL}$$
5. Determine the initial length of the TL_2_ line to obtain the assumed center frequency, neglecting the contribution of the vias:
  (14) $$l_{2}\approx \frac{c}{2f_{0}\sqrt{\epsilon_{reff}}}-2\left(l_{1}+\Delta l_{RL}\right)$$
6. Assume the radius of the via and calculate its equivalent inductance using (3).
7. Optimize by simulations the lengths and widths of the lines to achieve assumed filter specifications.
8. If step 7 fails, adjust the dimension of the radial line and go to step 1.

**Figure 15:**
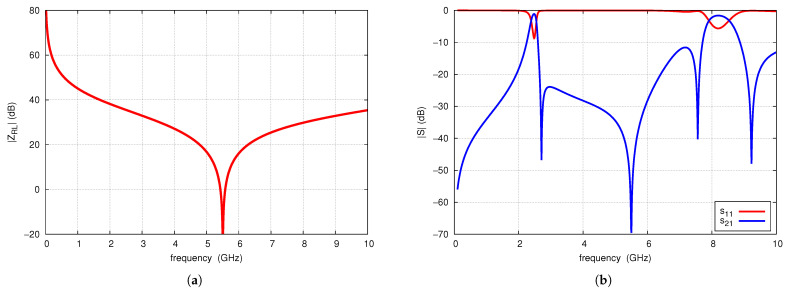
Radial line input impedance magnitude (**a**) and S-parameters of of the designed filter (**b**).

Using the algorithm presented, a filter has been designed with a nominal center frequency of 2.45 GHz, $f_{TZ1}$ is 2.7 GHz, and $f_{TZ_{RL}}$ is 5.5 GHz. Based on circuit simulations, the angle, radius and width of the connecting microstrip line of the radial lines are determined as 45°, 6.5 mm, and 0.4 mm, respectively. The modulus of input impedance $Z_{RL}$ of this line is shown in Figure 15a. Its value at the frequency $f_{TZ1}=2.7$ GHz is 50.3 Ω. On this basis, the equivalent length $\Delta_{RL}=7.32$ mm is calculated. Subsequently, it was found that the length of TL_1_ is found to be 9.8 mm, and the length of TL_2_ to be 3.5 mm. A via with a radius of 0.1 mm is selected, corresponding to an equivalent inductance of 0.227 nH. Circuit simulations of the filter designed in this manner are shown in Figure 15b. The filter exhibits a center frequency of 2.47 GHz, $f_{TZ1}=2.71$ GHz, and $f_{TZ_{RL}}=5.5$ GHz, but shows insufficient matching.

**Figure 16:**
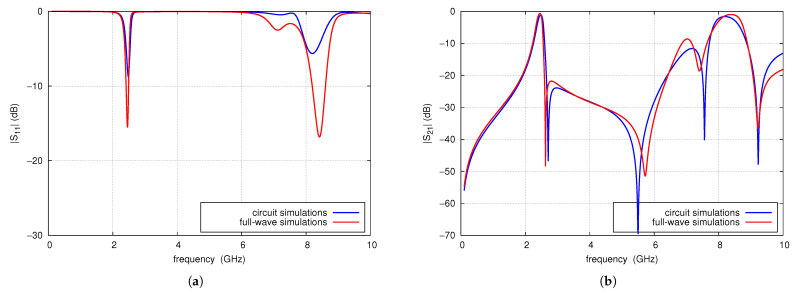
Comparison of circuit and full-wave simulation results of S-parameters of the designed filter: (**a**) $s_{11}$, (**b**) $s_{21}$.

Figure 16 compares the results of circuit simulations and full-wave simulations of the designed filter. A high level of consistency is observed between the results, with minor discrepancies primarily attributed to coupling between the open ends of the radial lines, which is not accounted for in the circuit simulations. Full-wave simulations demonstrate better impedance matching within the operating band. The center frequency of the filter is 2.45 GHz, which is identical in both simulation methods. The first transmission zero occurs at 2.62 GHz and the next at 5.71 GHz, with their frequencies differing slightly between the two simulations. The position and attenuation of the parasitic passbands are similar in both simulations. These passbands are separated by transmission zeros that occur at comparable frequencies; however, the attenuation levels predicted by the full-wave simulation are significantly lower. Despite some minor differences between the results, the proposed filter design algorithm performs satisfactorily in practice.

**Figure 17:**
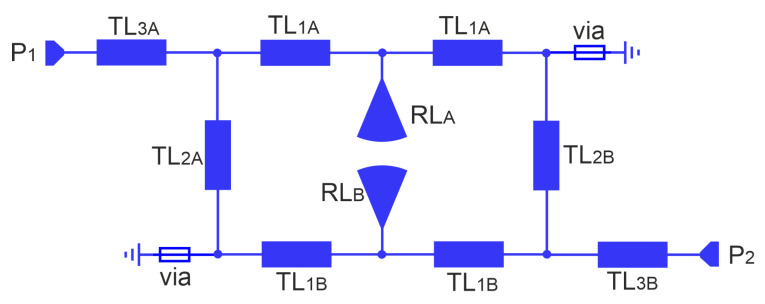
Equivalent circuit of the CMFDCDT filter.

Filter attenuation can be improved by adding high-impedance microstrip lines TL_3_ used to transform load and source impedances as shown in Figure 1 and Figure 17. Their operation is similar to that of a series inductor [37,38,39].

**Figure 18:**
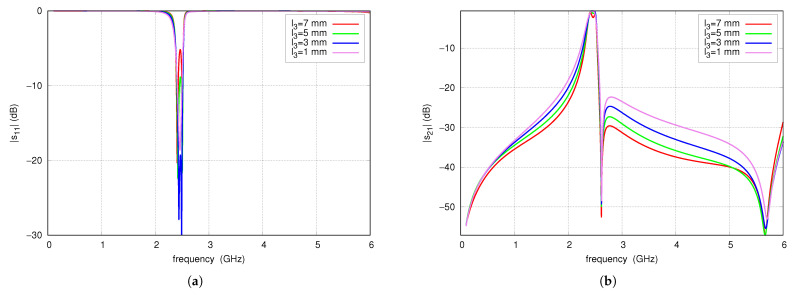
$s_{11}$ (**a**) and $s_{21}$ (**b**) of the CMFDCDT filter for four L_3_ lengths.

The effect of the TL_3_ line length on the S-parameters is presented in Figure 18 for TL_3_ impedance of 123 Ω. Varying the line length from 1 mm to 7 mm results in a noticeable increase in attenuation in the upper stopband, as well as a slight improvement in slope steepness in the lower transition band. An increase in line length reduces the influence of the load on the filter resonators that affect their matching and modifies the shape of the passband response. For a TL_3_ length of 7 mm, $s_{21}$ ripples of approximately 2 dB are observed. Considering the passband response, the attenuation in the stopband and the quolity of matching, a TL_3_ line length of 5 mm was selected as the satisfactory value.

### 4.1. Current Distribution

**Figure 19:**
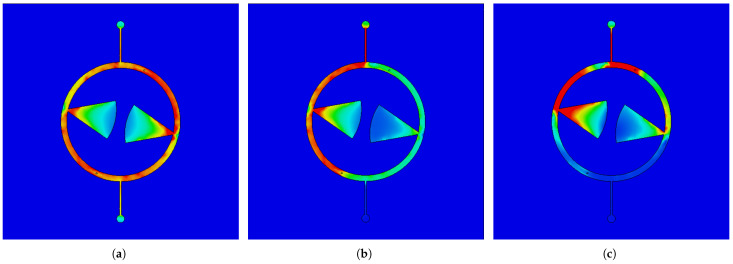
Current distribution within MFDCDT filter for frequencies: (**a**) 2.45 GHz, (**b**) 2.62 GHz, (**c**) 5.71 GHz.

For a better illustration of the filter operation, a full-wave analysis of the current distribution was performed. Figure 19 shows the current density for three characteristic frequencies. In all considered cases, the filter is fed from the upper port. Figure 19a shows the current distribution for the center frequency of the passband. The signal flows from the input to the output through both resonators. As can be seen, the current distribution in both resonators is similar in the corresponding lines TL_1_, TL_2_, RL and TL_3_. Figure 19b shows the current distribution for the first transmission zero frequency. The current in the TL_1_ and RL lines of the left (fed) resonator is greater than the current in the TL_1_ and RL lines of the right (coupled) resonator. The output line TL_3_ receives currents from the line TL_2_ of the left resonator and the line TL_1_ of the right resonator with similar amplitudes and opposite phases, causing compensation and resulting in strong output signal attenuation. Figure 19c shows the current distribution for the $f_{TZ_{RL}}$ frequency, at which the RL radial lines act as quarter-wave sections. As can be seen, the signal flowing through the TL_1_ lines is grounded by the RL radial lines RL. Furthermore, the currents flowing toward the TL_3_ line are mutually compensated.

### 4.2. Experimental Verification of Prototype of CMFDCDT Filter

**Figure 20:**
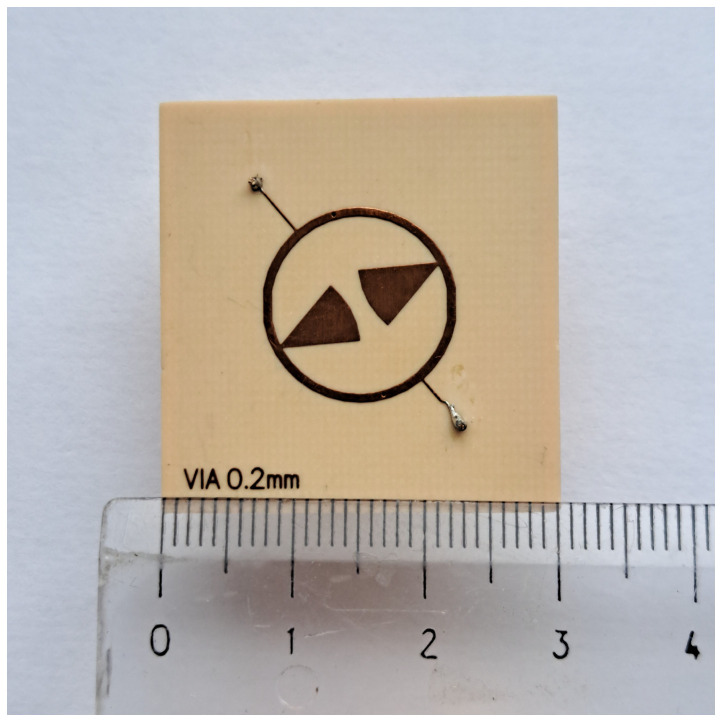
The photo of the CMFDCDT filter.

**Figure 21:**
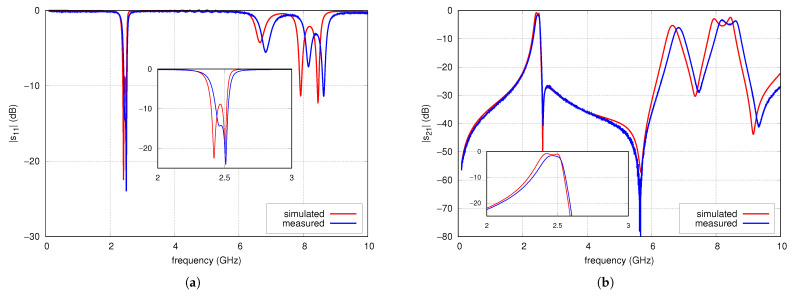
$s_{11}$ (**a**) and $s_{21}$ (**b**) parameters of CMFDCDT filter.

The prototype of the filter was fabricated using standard PCB technology is presented in Figure 20. Its parameters were measured using the PNA-L N5230A vector network analyzer. Figure 21 compares the measured and simulated frequency response of $s_{21}$ and $s_{11}$ of the filter in the range of 100 MHz to 10 GHz. The filter properties determined by measurements are given below. The center frequency is 2.458 GHz, the 3 dB bandwidth is 134 MHz, the insertion loss is 1.6 dB, and the $s_{11}$ is less than −10 dB in the range from 2.43 GHz to 2.52 GHz. The maximum out-of-band attenuation in the lower stopband is 50 dB at a frequency of 100 MHz and reaches 35 dB and 25 dB at frequencies of 0.9 GHz and 1.8 GHz, respectively. In the upper stopband, at a frequency of 2.63 GHz, there is the first transmission zero. Above 2.63 GHz, the attenuation throughout the stopband exceeds 30 dB, reaching more than 40 dB in the 4.7 to 5.9 GHz frequency range, where the following TZ, located at a frequency of 5.633 GHz, has an effect. Above this frequency, the filter attenuation decreases monotonically, reaching 20 dB at 6.43 GHz. Next, in the filter characteristics at frequencies of 6.835 GHz and 8.4 GHz, there are parasitic passbands with attenuations of 6.1 and 3.4 dB, respectively. Above the second parasitic passband, the filter attenuation increases to 41 dB at 9.32 GHz and then decreases to 27 dB. The filter introduces attenuation of the third harmonic of the nominal frequency 2.465 GHz by approximately 28 dB. The simulation results differ slightly from the measurement results. The center frequency is 2.45 GHz, the TZs frequencies are 2.62 GHz and 5.71 GHz and both the measured and the simulated characteristics in the stopband are practically identical up to 4.5 GHz. Above 4.5 GHz, the two characteristics remain very similar, with differences resulting from a slight frequency shift relative to each other.

## 5. Discussion

Table 1 compares the properties of seven microstrip reference filters found in the literature that operate in the ISM 2.4 GHz frequency band with those of the filter presented in this work. The following parameters were considered: relative bandwidth (RBW), insertion loss (IL), number of transmission zeros (NTZ), number of resonators (NR), number of layers (NL), electrical size and attenuation occurring in the stopband at the operating frequencies of radio communication systems such as cellular and Wi-Fi networks. Most comparable filters exhibit a relative bandwidth of 4% to 6%, with the exception of filters [30,31], which demonstrate significantly wider bandwidths of 24.6% and 35%, respectively. The relative bandwidth of the proposed filter is 5.8%. All filters exhibit insertion losses in the range of 1 to 2.17 dB. The insertion loss of proposed filter is 1.6 dB. All $s_{21}$ characteristics are shaped by transmission zeros located in the transition or stopbands. The number of applied transmission zeros ranges from 2 to 9 [36] while the number of resonators is 2 or 4. The proposed CMFDCDT filter adopts 2 resonators and 2 TZs. Most filters are implemented on two-layer substrates (such as the proposed filter), with the exception of filters [30,36], which are implemented on three- and eight-layer substrates, respectively. The smallest area occupied by the compared filters is 0.0256$\lambda^{2}$ for the filter [30], which was manufactured on a three-layer substrate, while the two-layer filters occupy approximately 0.06$\lambda^{2}$, except for the filter [31], which has the largest area of 0.41$\lambda^{2}$. The area of the proposed CMFDCDT filter is 0.064$\lambda^{2}$. The out-of-band properties of the filters are compared in selected bands. Subbands 0.9 GHz, 1.8 GHz and 2.1 GHz are chosen for the lower stopband. Subbands 3.5 GHz i U-NII 5 GHz are chosen for the upper stopband. When comparing the out-of-band $s_{21}$ characteristics, it was taken into account that filters [17,27] are dual-band, while filters [30,31] exhibit such wide passbands that they simultaneously cover the 1.8 GHz and 2.1 GHz bands. The attenuation values for these specific bands are omitted for [17,27,30,31] in the table and are indicated as ‘-’. Most filters provide out-of-band attenuation greater than 20 dB. The highest attenuation in the upper and lower stopbands is achieved by the quadruple-tuned filters [29,30], reaching up to 65 dB. For double-tuned filters in the lower stopband, the highest attenuation is achieved by [16], which is 37 dB while the lowest attenuation exhibited by the proposed filter is 18 dB for 2.1 GHz band. In the upper stopband, the proposed filter achieves the highest attenuation among all exceeding 32 dB. A comparison of the proposed filter with other double-tuned, two-layer filters shows that its electrical dimensions, bandwidth, and insertion loss are similar. The proposed filter is among those with the smallest number of transmission zeros. However, it achieves the highest attenuation in the upper stopband among the compared double-tuned filters, owing to the effective positioning of the one of the TZ. In the lower stopband, the proposed network provides attenuation comparable to its competitors, despite the lack of TZs in this band.

## 6. Conclusions

The article presents a compact double-tuned microstrip fixed-frequency double-coupled filter with selected band suppression. The developed filter is suitable as an input filter for receivers used in wireless IoT sensors. The frequency response of the filter is shaped by two transmission zeros. The first transmission zero is located in the upper passband, whereas the next is located at the selected frequency of the upper stopband. A reduction in the electrical dimensions of the filter was achieved by employing two radial lines as resonator components. A simple design method for this type of filter is introduced, and its effectiveness is demonstrated with an example filter designed for the 2.4 GHz ISM band, with simultaneous suppression of the U-NII 5 GHz band. The performance of the proposed filter is compared with competitor filters reported by other authors. Despite the simplicity of the proposed design, it offers superior attenuation in the upper stopband and facilitates effective band suppression through precise positioning of the TZs enabled by the application of the radial lines. The layout area of the proposed filter is comparable to that of competing designs, although there remains room for further optimization. Future work will focus on reducing the occupied area without compromising electrical performance, as well as extending the design methodology to higher-order filters based on the described operating principle.

## Figures and Tables

**Table 1:** Comparison between the proposed and the reference filters.

							Attenuation(dB) @ f(GHz) ^1^					Size ^2^
Ref	f(GHz)	RBW (%)	IL (dB)	NTZ	NR	NL	0.9	1.8	2.1	3.6	5	$\left(\lambda_{g}^{2}\right)$
[29]	2.5	4	1.2	9	4	2	44	35	35	45	43	0.0594
[16]	2.5	4	1.05	4	2	2	37	32	35	30	32	0.0784
[30]	2.4	24.6	1.66	2	4	3	65	20	-	7	44	0.0256
[36]	2.45	4	1.7	2	2	8	20	18	7	9	24	– ^3^
[31]	2	35	1	6	4	2	33	-	-	30	15	0.41
[27]	2/2.5	5.3/5.8	2.17/1.71	3	2	2	35	9	-	24	12	0.0544
[17]	1.8/2.4	8.9/7.4	1.08/1.42	3	2	2	29	-	24	25	20	0.04
This work	2.47	5.8	1.6	2	2	2	35	25	18	32	43	0.064

^1^ The frequencies denotes the following bands: 0.9: (890–960 MHz), 1.8: (1710–1880 MHz), 2.1: (1920–2170 MHz), 3.6: (3.4–3.8 GHz), 5: (5.15–5.825 GHz). ^2^ Calculated for central frequency. ^3^—Multimaterial. RBW —Relative bandwidth, IL—Insertion loss, NTZ—Number of transmission zeros, NR—Number of resonators, NL—Number of layers.

## Data Availability

Data are contained within the article.

## Abbreviations

The following abbreviations are used in this manuscript:

RF	Radio Frequency
VHF	Very High Frequencys
U-NII	Unlicensed National Information Infrastructure
ISM	Industrial, Scientific, and Medical
SIR	Stepped-Impedance Resonators
OLR	Open-Loop Resonator
TZ	Transmission Zero
CMFDCDT	Compact Microstrip Fixed-frequency Double-Coupled Double-Tuned
SAW	Surface Acoustic Wave
PCB	Printed Circuit Board
RBW	Relative Bandwidth
IL	Insertion Loss
NTZ	Number of Transmission Zeros
NR	Number of Resonators
NL	Number of Layers
IoT	Internet of Things
